# Oxidative Status and Histological Evaluation of Wild Boars’ Tissues Positive for Zearalenone Contamination in the Campania Region, Southern Italy

**DOI:** 10.3390/antiox12091748

**Published:** 2023-09-10

**Authors:** Sara Damiano, Consiglia Longobardi, Gianmarco Ferrara, Nadia Piscopo, Lorenzo Riccio, Valeria Russo, Valentina Meucci, Lucia De Marchi, Luigi Esposito, Salvatore Florio, Roberto Ciarcia

**Affiliations:** 1 Department of Veterinary Medicine and Animal Productions, University of Naples “Federico II”, 80137 Napoli, Italy; sara.damiano@unina.it (S.D.); consiglia.longobardi@unina.it (C.L.); gianmarco.ferrara@unina.it (G.F.); lorenzo.riccio@unina.it (L.R.); valeria.russo@unina.it (V.R.); luigi.esposito4@unina.it (L.E.); florio@unina.it (S.F.); rciarcia@unina.it (R.C.); 2 Department of Veterinary Science, University of Pisa, 56122 Pisa, Italy; lucia.demarchi@unipi.it

**Keywords:** Zearalenone, wild boar, oxidative stress, liver, kidney, muscle

## Abstract

Zearalenone (ZEN) is a mycotoxin produced by fungi belonging to the genera *Fusarium* spp. and commonly found in feed and food. It is frequently related to reproductive disorders in farm animals and, occasionally, to hyperestrogenic syndromes in humans. Nowadays, knowledge about ZEN effects on wild boars (*Sus scrofa*) is extremely scarce, despite the fact that they represent one of the most hunted game species in Italy. The aim of this study was to investigate how ZEN affects the liver, kidney, and muscle oxidative status and morphology of wild boars hunted in various locations throughout the province of Avellino, Campania Region, Southern Italy, during the 2021–2022 hunting season. Superoxide dismutase (SOD), catalase (CAT), and glutathione peroxidase (GPx) activities, as well as the malondialdehyde (MDA) levels, were assessed by colorimetric assays; tissue morphology was evaluated by hematoxylin–eosin and Masson’s stains. Our data showed that ZEN contamination might result in oxidative stress (OS) and some histopathological alterations in wild boars’ livers and kidneys rather than in muscles, emphasizing the importance of developing a wildlife monitoring and management strategy for dealing not only with the problem of ZEN but the surveillance of mycotoxins in general.

## 1. Introduction

Zearalenone (ZEN) is an estrogenic mycotoxin produced by filamentous fungi belonging to *Fusarium* spp., mainly *F. culmorum* and *F. graminearum* [1]. Due to its global dissemination and economic impact, it poses a risk to animal and human health. It is well established that ZEN induces hormonal imbalances in several animal species and, in some cases, has been linked to breast cancer in humans [2,3]. This mycotoxin accumulates in a large variety of livestock feeds, such as corn and other grains, dried fruits, and spices, particularly during high temperature and humidity periods [4]. In addition, its thermostability allows it to withstand storage, milling, processing, and distribution [5]. Moreover, it accumulates in cereals mainly before and after harvest in poor storage conditions [6]. A recent epidemiological study has shown that ZEN is a major mycotoxin found in feed and complete feed for animals [7]. After oral administration, ZEN is metabolized in the liver of monogastric animals and is mainly biotransformed into hepatocytes in the presence of reducing factors such as nicotinamide adenine dinucleotide phosphate (NADPH), which leads to the transformation of ZEN into α-zearalenol (α-ZEL), β-zearalenol (β-ZEL), α-zearalanol (α-ZAL), and β-zearalanol (β-ZAL). These metabolites are then conjugated with sulfonic or glucuronic acid and eliminated mainly via urine in pigs [8]. Even though ZEN metabolites have different affinities for estrogen receptors ERα and ERβ [9], they can disrupt endocrine functions in various animal species in both sexes [10] and are also immunotoxic [11], hepatotoxic [12], hematotoxic [13], and nephrotoxic [14]. A key mediator of the harmful effects induced by ZEN exposure is thought to be oxidative damage [15,16]. Several studies have revealed that oxidative stress (OS) is a significant component for ZEN toxicity both in vivo and in vitro [17], although the exact harmful mechanism is still unclear. Moreover, in livestock and poultry, ZEN contamination in food and feeds is ruled and managed according to the European Commission (EU No. 2023/915), while no limits are set in the case of wildlife and game meat consumption.

In recent times, several factors, such as the absence of natural predators, rural depopulation, and expansion of forest areas, have favored a widespread intensifying of wild boar densities. The wild boar (*Sus scrofa*) is the most hunted wildlife species in Italy. It is an opportunistic, omnivorous mammal, which can vary its diet and traverse considerable distances in a day. Due to its remarkable capacity to adapt to a wide variety of habitats (from plains to mountains), wild boar feed on any resources available in the natural environment [18,19], even cereals susceptible to contamination by ZEN [20]. A Poland study demonstrated that the prevalence of ZEN in wild boars’ organs mainly depends on habitat type, and the presence of extensive maize fields increases the contamination [21]. Therefore, due to its eating habits, wild boar may serve as an environmental bioindicator not exclusively for infectious diseases [22,23] but also as natural pollutants such as mycotoxins [24,25], in addition to being used for food and sport hunting. Since game meat consumption is a common practice, any contamination, including that induced by mycotoxins, could be considered a public health risk. Among mycotoxins, ZEN represents a global health and economic challenge, since its rate is estimated to increase due to climate change [26]. At the same time, the increase in natural wild boar populations has stimulated interest in this species as a meat producer and sentinel for environmental contaminants [27]. Recent findings revealed ZEN contamination in wild boars hunted in the province of Avellino, an area of the Campania Region with the highest number of these wild animals [28]. Since ZEN effects in wild boars are poorly documented and mainly focus on its detrimental effects on reproductive organs, the purpose of this study was to investigate the effect of ZEN on some parameters of OS and morphologic alterations in the liver, kidney, and muscle of ZEN-contaminated wild boars hunted in the province of Avellino, which organs are used in a variety of traditional recipes, including stews, sauces, cured meats, and sausages.

## 2. Materials and Methods

### 2.1. Ethics Statement

For hunter-harvested boars, no approval was needed from an ethics committee, since the animals were not culled for research purposes. These animals were legally hunted during the wild boar hunting season, from November 2021 to February 2022, in different areas of the province of Avellino, Campania Region, Southern Italy. They were hunted by authorized hunters during the 2021–2022 hunting season, approved by the local authorities. 

### 2.2. Sample Collection

Our sampling consisted of 34 livers, 21 muscles (diaphragm), and 12 kidneys that resulted positive for ZEN contamination by HPLC-FLD analysis in our previous study [28] and were grouped into the Zearalenone-positive group (ZEN+). A total of 14 wild boar samples negative to ZEN for each organ (14 livers, 14 diaphragm, and 14 kidneys) were assigned to the Zearalenone-negative group (ZEN−). In particular, the mean concentrations ± standard error (SE) of ZEN were 1.71 ± 0.339 ng/g in the liver, 1.49 ± 0.493 ng/g in the muscle, and 0.65 ± 0.260 ng/g in the kidney (limit of detection (LOD) = 0.05 µg/kg for each organ), as reported by Longobardi et al. [28].

Liver, kidney, and skeletal muscle (diaphragm) samples were collected from each wild boar. One aliquot was immediately frozen at −80 °C to perform an enzymatic activities evaluation, and another was preserved in 10% neutral-buffered formalin to carry out histological investigations.

### 2.3. Enzyme Activities Quantification and Malondialdehyde (MDA) Assay

One gram of each liver, kidney, and muscle sample was rinsed with phosphate-buffered saline (PBS) to remove any blood cells and clots before being divided into 4 aliquots, homogenized on ice with an electric tissue homogenizer (Tissue Lyser, Qiagen, Milano, Italy), and centrifuged at different speeds depending on the test. 

The resulting supernatants were kept at −80 °C until testing and were used to evaluate, colorimetrically, the SOD, CAT, and GPx activities using a spectrophotometer, as described by Rudolph et al. [29]. 

SOD activity was assessed using the Superoxide Dismutase Assay Kit (Cayman Chemical, Ann Arbor, MI, USA). Briefly, samples, after homogenization (20 mM HEPES buffer, pH 7.2, containing 1 mM EGTA, 210 mM mannitol, and 70 mM sucrose per gram tissue), were centrifuged at 1500× *g* for 5 min at 4 °C and, finally, incubated for 30 min at room temperature with the xanthine oxidase reagent, according to the manufacturer’s instructions. Absorbance was measured at 460 nm using a spectrophotometer (Glomax Multi detection system, Promega, Milano, Italy), and the results were expressed as U/mL. 

CAT activity was quantified by the Catalase Assay Kit (Cayman Chemical, Ann Arbor, MI, USA). After homogenization (50 mM potassium phosphate, pH 7.00, containing 1 mM EDTA per gram tissue), each sample was centrifuged at 10,000× *g* for 15 min at 4 °C and assayed according to the manufacturer’s instructions. Absorbance was read at 540 nm using a spectrophotometer (Glomax Multi detection system, Promega, Milano, Italy), and the results were expressed as nmol/min/mL.

GPx activity was measured by the Gluthatione Peroxidase Assay Kit (Cayman Chemical, Ann Arbor, MI, USA). Sample homogenization (50 mM Tris-HCl, pH 7.5, 5 mM EDTA, and 1 mM DTT per gram tissue) was followed by centrifugation at 10,000× *g* for 15 min at 4 °C. The resulting samples were then tested according to the manufacturer’s instructions, the absorbance was read at 340 nm using a spectrophotometer (Glomax Multi detection system, Promega, Milano, Italy), and the results were expressed as nmol/min/mL. 

Malondialdehyde (MDA), a marker of lipid peroxidation, was calculated according to Gassó et al. [30]. The optical density (OD) of the supernatants containing MDA and forming the MDA–TBA (thiobarbituric acid) adduct was read using a spectrophotometer (Glomax Multi detection system, Promega, Milano, Italy) at a wavelength of 535 nm. Data were expressed in nmol MDA/mL.

Enzymatic activities and MDA assays were performed on 10 livers, muscles, and kidneys belonging to the ZEN- group and on 34 livers, 12 kidneys, and 21 muscles belonging to the ZEN+ group.

### 2.4. Histopathological Studies

Samples of kidney, liver, and muscle aliquots fixed in 10% neutral-buffered formalin solution for 48 h were dehydrated in ethyl alcohol and embedded in paraffin. Four-micrometer sections were stained with hematoxylin and eosin and Masson’s trichrome stain, then examined and photographed with a light microscope (Nikon Eclipse E600, Tokyo, Japan) associated with a microphotography system Nikon digital camera (DMX1200). 

The ZEN- group included 14 samples of liver, kidney, and skeletal muscle; the ZEN+ group included 13 livers, 9 kidneys, and 9 skeletal muscles positive for ZEN. A histological scoring system was used considering the most representative lesions in the kidneys, livers, and muscles.

In the kidneys, the hematoxylin- and eosin-stained sections were used to score the severity of inflammation and the presence of proteinaceous material in the Bowman’s spaces and tubules lumen. Kidney lesions were scored by evaluating at least 10 microscopic fields at 20× magnification and using already defined scoring systems. Notably, inflammation was scored as follows: score 0, no inflammatory foci; score 1 (mild), <2 foci per 20× field; score 2 (moderate), 2–4 foci per 20× field; and score 3 (severe), >4 foci per 20× field. The presence of proteinaceous material in the Bowman’s spaces and tubules lumen were scored from 0 to 3 (0 = absent; 1 = mild; 2 = moderate; 3 = severe) [31]. Lastly, the severity of the fibrosis was evaluated on the Masson’s trichrome-stained section based on the ratio between fibrosis and the total area examined observing 10 fields at 20× into the following categories: 0 (absent), 1 (mild; <10%), 2 (moderate; 10–30%), and 3 (severe; >30%).

Hepatic and skeletal muscle lesions were scored by evaluating at least 10 microscopic fields at 20× magnification and using already defined scoring systems. Inflammation was scored as follows: score 0, no inflammatory foci; score 1 (mild), <2 foci per 20× field; score 2 (moderate), 2–4 foci per 20× field; and score 3 (severe), >4 foci per 20× field. The extent of degeneration was scored as follows: score 0, < 5% of hepatocytes; score 1 (mild), 5–33%; score 2 (moderate), >33–66%; and score 3 (severe), >66% [32]. The severity of the fibrosis was evaluated based on the ratio between fibrosis and the total area examined observing 10 fields at 20× into the following categories: 0 (absent), 1 (mild; <10%), 2 (moderate; 10–30%), and 3 (severe; >30%) [33].

### 2.5. Statistical Analysis

Statistical analysis of the enzymatic activities and lipid peroxidation was expressed as the mean ± standard deviation (SD) of experiments performed in triplicate. Statistical analyses were performed using GraphPad (version 8.0; GraphPad Software Inc., San Diego, CA, USA). The Shapiro–Wilk test and Kolmogorov–Smirnov test were used to determine the normality of the data distribution. Differences between the ZEN− and ZEN+ groups were evaluated using an unpaired *t*-test, while differences among the means of each histological semiquantitative score were evaluated using the Mann–Whitney *U* test. Values of *p* < 0.05 were considered significant.

## 3. Results

### 3.1. The Effect of ZEN on Lipid Peroxidation

**Figure 1:**
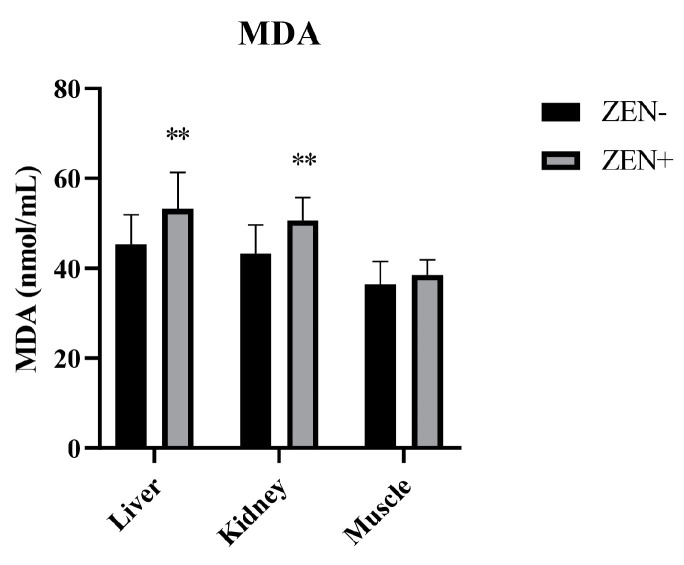
Effect of Zearalenone (ZEN) on lipid peroxidation measured by the malondialdehyde (MDA) assay in wild boars’ livers (*n* = 34 ZEN+; *n* = 10 ZEN−), kidneys (*n* = 12 ZEN+; *n* = 10 ZEN−), and muscles (*n* = 21 ZEN+; *n* = 10 ZEN−). Zearalenone-negative group (ZEN−); Zearalenone-positive group (ZEN+). The results are expressed as the mean ± standard deviation (SD). ** *p* < 0.01 vs. ZEN−. Data are expressed in nmol of MDA per mL.

The MDA levels in the liver and kidney tissues were significantly increased in the Zearalenone-positive samples (ZEN+) compared to the ZEN-uncontaminated ones (ZEN−). The values elevated from 45.3 ± 6.6 (ZEN−) to 53.2 ± 8.1 (ZEN+) in the liver and from 43.2 ± 6.4 (ZEN−) to 50.6 ± 5.1 (ZEN+) in the kidney, thus resulting in a statistical significance of *p* < 0.01 in both cases. Indeed, no statistical changes in the MDA levels at the muscle tissue level were detected. In fact, the MDA value in the muscle tissue was 36.4 ± 5.12 in the ZEN+ compared to 38.5 ± 3.4 in the ZEN− group (Figure 1).

### 3.2. SOD, CAT, and GPx Activities Alterations upon ZEN Exposure

**Figure 2:**
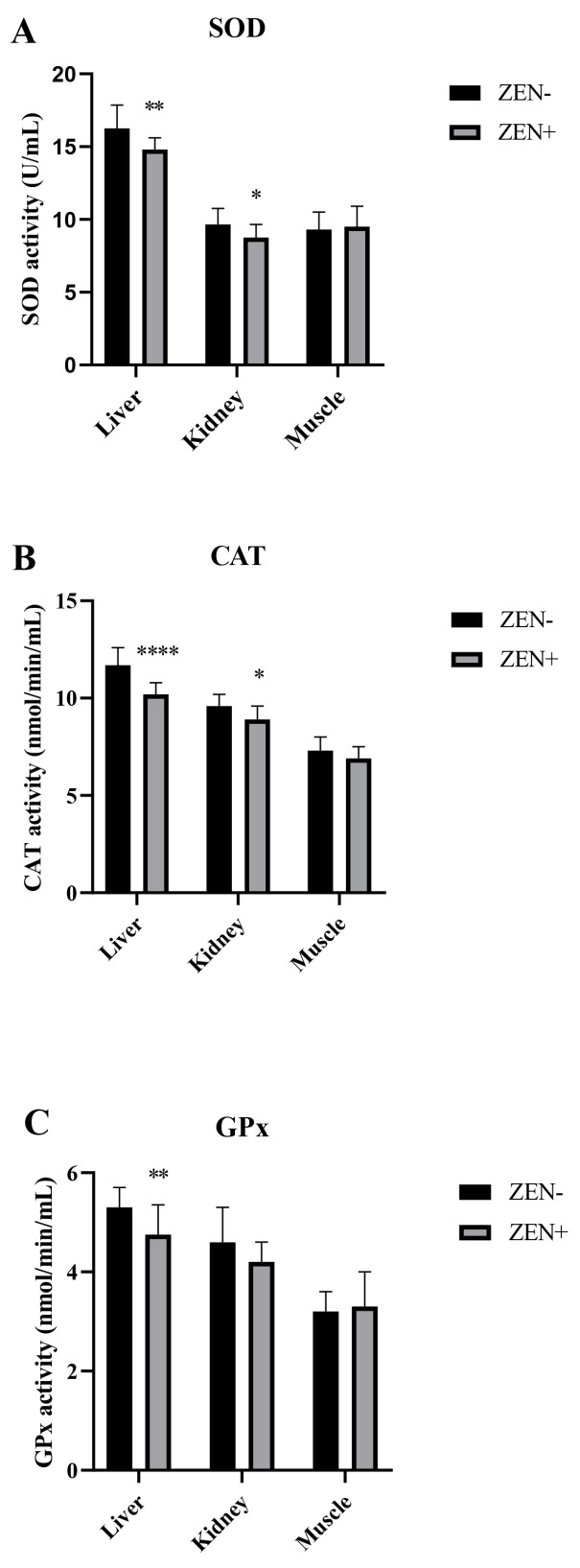
Effects of Zearalenone (ZEN) on (**A**) superoxide dismutase (SOD), (**B**) catalase (CAT), and (**C**) glutathione peroxidase (GPx) activities in wild boars’ livers (*n* = 34 ZEN+; *n* = 10 ZEN−), kidneys (*n* = 12 ZEN+; *n* = 10 ZEN−), and muscles (*n* = 21 ZEN+; *n* = 10 ZEN−). Zearalenone-negative group (ZEN−); Zearalenone-positive group (ZEN+). The results are expressed as the mean ± standard deviation (SD). * *p* < 0.05, ** *p* < 0.01, and **** *p* < 0.001 vs. ZEN−. Data are expressed as U/mL for SOD and as nmol/min/mL for CAT and GPx.

The antioxidant markers SOD, CAT, and GPx in the liver, kidney, and muscle tissues of wild boars are shown in Figure 2A–C. The activities of SOD and CAT were decreased significantly in the liver and kidney of wild boars belonging to the ZEN+ group when compared to the ZEN− one. In fact, the SOD values decreased from 16.30 ± 1.60 (ZEN−) to 14.8 ± 0.80 (ZEN+) in the liver (** *p* < 0.01) and from 9.65 ± 1.10 (ZEN−) to 8.75 ± 0.90 (ZEN+) in the kidney (* *p* < 0.05) (Figure 2A). The CAT values reduced from 11.7 ± 0.9 (ZEN−) to 10.2 ± 0.60 (ZEN+) in the liver (**** *p* < 0.0001) and from 9.6 ± 0.61 (ZEN−) to 8.9 ± 0.70 (ZEN+) in the kidney (* *p* < 0.05) (Figure 2B). The GPx values shifted from 5.30 ± 0.4 (ZEN−) to 4.2 ± 0.40 (ZEN+) in the liver (* *p* < 0.01) (Figure 2C). No significant variations were detected in the kidney GPx activity, with a shifting trend from 4.60 ± 0.70 (ZEN−) to 4.20 ± 0.40 (ZEN+). The SOD, CAT, and GPx activities in the muscle tissues of ZEN-positive wild boars (ZEN+) did not differ statistically among the groups, and the values shifted from 9.30 ± 1.20 to 9.50 ± 1.40 for SOD, 7.30 ± 0.70 to 6.90 ± 0.60 for CAT, and 3.20 ± 0.40 to 3.30 ± 0.70 for GPx in the ZEN− and ZEN+ groups, respectively.

### 3.3. Liver, Kidney, and Muscle Histopathological Examination

**Figure 3:**
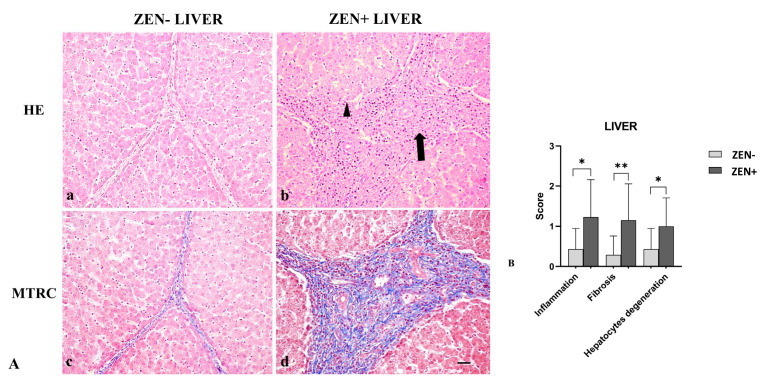
(**A**) Wild boar, liver, hematoxylin–eosin (HE) (**a**,**b**), and Masson’s trichrome (MTRC) stains (**c**,**d**), 20× magnification, scale bar = 50 µm. Zearalenone-negative group (ZEN−) (**a**,**c**) and Zearalenone-positive group (ZEN+) (**b**,**d**). Livers of the ZEN− group (*n* = 14) showed only a few disseminated swollen hepatocytes (**a**) and a normal amount of interstitial connective tissue (blue) with MTRC stain (**c**). Livers of the ZEN+ group (*n* = 13) showed moderate, periportal lymphocytes inflammatory infiltrate (**arrow**) and numerous disseminated swollen hepatocytes (**arrowhead**) (**b**). The ZEN+ group also showed portal spaces moderately expanded by fibrous connective tissue (blue) with MTRC stain (**d**). (**B**) Severity scores for inflammation, fibrosis, and hepatocytes degeneration. * *p* < 0.05, and ** *p* < 0.01 vs. ZEN−.

Livers of the ZEN− group did not show significant pathologic findings; rarely (only in six samples), sparse infiltrating inflammatory cells were evident, and few disseminated hepatocytes showed swollen cytoplasm; fibrosis was observed in four cases. Differently, livers from the ZEN group showed mild-to-moderate, multifocal, and periportal lymphoplasmacytic inflammation and numerous swollen hepatocytes associated with moderate interlobular and periportal fibrosis (Figure 3A).

Based on histopathology scoring, the ZEN+ group showed more severe inflammation and degeneration of hepatocytes (* *p* < 0.05) compared to the ZEN− group. Fibrosis was more frequently observed in all cases of ZEN+ (** *p* < 0.01) when compared to ZEN− animals (Figure 3B).

**Figure 4:**
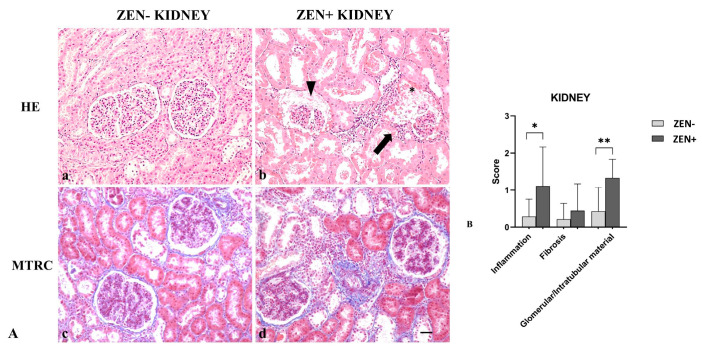
(**A**) Wild boar, kidney, hematoxylin–eosin (HE) (**a**,**b**), and Masson’s trichrome (MTRC) stains (**c**,**d**), 20× magnification, scale bar = 50 µm. Zearalenone-negative group (ZEN−) (**a**,**c**); ZEN-positive group (ZEN+) (**b**,**d**). Kidneys of the ZEN− group (*n* = 14) occasionally showed a small amount of intratubular proteinaceous material (**a**) and a mild amount of interstitial connective tissue (blue) with MTRC stain (**c**). Kidneys of the ZEN+ groups (*n* = 9) showed segmental necrosis of the glomerulus (**asterisk**), focal interstitial lymphoplasmacytic inflammatory infiltrate (**arrow**), and abundant proteinaceous material in the Bowman’s spaces and tubules lumen (**arrowhead**) (**b**). Kidneys of the ZEN+ group showed the interstice moderately expanded by fibrous connective tissue (blue) with MTRC stain (**d**). (**B**) Severity scores of inflammation, fibrosis, and presence of proteinaceous material in the Bowman’s spaces and tubules lumen. * *p* < 0.05 and ** *p* < 0.01 vs. ZEN−.

Kidneys from the ZEN− group appeared normal, with a mild accumulation of intratubular proteinaceous material (5 out 14). Only in one out of three cases, we observed mild and scattered lymphocytic infiltration in the interstitial space and mild fibrosis. Instead, kidneys of the ZEN+ group showed a multifocal interstitial inflammatory infiltrate. The Bowman’s space and tubules lumen contained abundant proteinaceous material, with a reduction of the Bowman’s space and glomerular atrophy. Tubular epithelial cells were often atrophic or degenerate. Fibrosis was absent except for three cases, where it was mild in comparison to the ZEN− group (Figure 4A).

Masson’s trichrome (MTRC) stain showed no significant differences in the fibrotic fibers in the ZEN+ group compared to the ZEN− group. The ZEN+ group showed moderate inflammation (* *p* < 0.05) and the presence of proteinaceous material (** *p* < 0.01) compared to the ZEN− group (Figure 4B).

Wild boar skeletal muscles from the ZEN− group did not show significant pathological changes. Rarely, a mild inflammatory infiltrate and mild fibrosis (only 2 out of 14 cases) were observed. 

**Figure 5:**
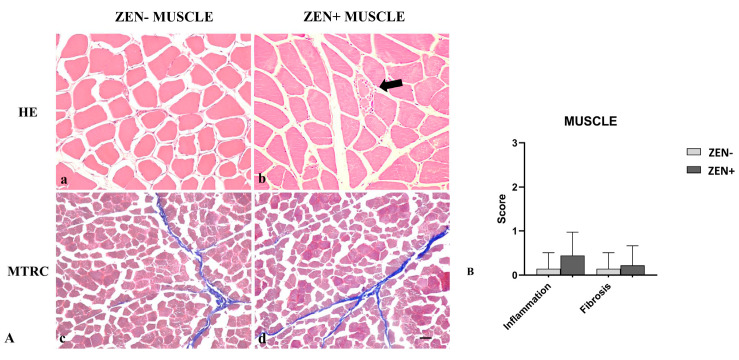
(**A**) Wild boar, skeletal muscle, hematoxylin–eosin (HE) (**a**,**b**), and Masson’s trichrome (MTRC) stains (**c**,**d**), 20× magnification, scale bar = 50 µm. Zearalenone-negative group (ZEN−) (**a**,**c**); Zearalenone-positive group (ZEN+) (**b**,**d**). Skeletal muscle of the ZEN− group (*n* = 14) did not show significant pathological changes in the muscle fibers and amount of collagen (**a**,**c**). Muscles of the ZEN+ group (*n* = 9) showed mild variability in the muscle fiber diameter and necrotic fibers with a focal area of inflammation (**arrow**) (**b**). MTRC stain showed a normal content of collagen tissue (**d**). (**B**) Severity scores of inflammation, and fibrosis.

Differently, the morphologic assessment of muscle tissues from the ZEN+ group showed mild variability in the myofiber diameter, fibers reduced in size (atrophy) with an angular profile, and fibers with pale sarcoplasm and optically empty vacuoles (degeneration). Sporadic necrotic muscle fibers were surrounded and infiltrated by a moderate number of reactive macrophages and fewer lymphocytes and plasma cells. In a few cases, four out of nine, the presence of mild lymphoplasmacytic inflammatory infiltrate was observed in skeletal muscles. Mild fibrosis was observed only in two cases (Figure 5A).

Based on histopathology scoring, the severity of the inflammation and fibrosis of the ZEN+ group cases were not statistically significant compared to the ZEN− ones (Figure 5B).

## 4. Discussion

Correlation between ZEN and its toxicity in wild boars is scarce, and the literature focuses on its toxicity toward reproductive organs [34] because of the well-known ZEN mycoestrogenity. Since the increase in natural wild boar populations has stimulated interest in this species as a meat producer, it is deemed necessary to investigate the toxicity of ZEN in edible tissues, such as the muscles, livers, and kidneys, of this wild animal. In particular, this study focused on the province of Avellino because of its highest percentage of this animal species in the Campania Region.

In this study, the impact of ZEN on the oxidative state of wild boars (*Sus scrofa*) was evaluated. Precisely, it examined the effect of ZEN on some parameters of OS in the livers, kidneys, and muscles of wild boars detected as positive for ZEN contamination in a previous work [19].

It is well known that the toxic effect of mycotoxins can lead to OS and to the production of free radicals [35]. The increase in free radicals leads to a malfunction of the antioxidant system with damage to the DNA, proteins, and lipids [36]. In this regard, antioxidant enzymes play pivotal roles in eliminating excess radical oxygen species (ROS) maintaining cellular environmental homeostasis. The analysis performed in this work focused on the MDA levels and SOD, CAT, and GPx activities in wild boars’ livers, kidneys, and muscles. The results showed that MDA was clearly enhanced in the ZEN-positive group, evidencing a lipid peroxidation increase, in accordance with some in vivo experiments conducted on mice [37] and piglets [38]. 

Regarding enzyme activity, a decreased activity of SOD, CAT, and GPx in both the livers and kidneys of the ZEN+ group suggests the induction of OS in these organs. In addition, the increase in MDA as a metabolite of the lipid peroxidation process is directly linked to the reduction of the enzymatic antioxidant activity of the tested enzymes in response to ZEN contamination [39]. The oxidative imbalance condition in ZEN-contaminated wild boars’ livers and kidneys also compromise their morphological characteristics, confirming a possible long-term exposure to ZEN and a consequent chronic effect. In particular, the most severe effects were highlighted in the liver, which impairment induced by ZEN is documented to be associated with OS [40,41]. In fact, significant fibrosis with the evidence of hepatocyte degradation can be observed in the ZEN+ group’s livers. These findings are consistent with previous studies from the literature in which liver lesions and alterations of some enzymatic indices of the hepatic function were detected in rats [41], rabbits [42], and gilts [43], as well as Polish wild boars [44], after ZEN exposure. Moreover, hepatocyte degeneration found in the livers supports the notion that ZEN is a potent apoptosis inductor in the mammalian system in a dose- and time-dependent manner, as previously reported by in vivo and in vitro experiments [45,46]. 

The histologic examination of the kidneys of wild boars belonging to the ZEN+ group revealed no evidence of fibrotic tissue accumulation. However, the presence of abundant proteinaceous material in the Bowman’s space and tubules lumen and multifocal interstitial inflammatory infiltrate, on the other hand, indicated that the inflammation pathway was activated. ZEN’s metabolism, mostly hepatic (as in the case of pigs [47]), and the absence of renal fibrosis, as well as the small sample size, could explain the nonsignificant variation in GPx enzymatic activity in the kidneys. In fact, in a previous study, in wild boars’ kidneys, α-ZEL accumulation was found [28], which activity was more estrogenic [48] than nephrotoxic. In this regard, it could be interesting to examine the kidney tissues positive for α-ZEL and compare their relative nephrotoxicity [37].

Wild boar exposure to ZEN did not significantly influence the activities of the tested antioxidant enzymes in the muscles, and not even lipid peroxidation was affected. This is consistent with the histologic data, since no significant morphological alterations or signs of fibrosis in the muscles of ZEN-contaminated wild boars have been observed. This evidence fits with the findings of Oliver et al., whose work showed that ZEN do not regulate skeletal muscle protein synthesis in prepubertal gilts [49]. As a result, ZEN distribution in wild boars could have no effects on the edibility of the animal muscles. However, these tissues are polluted, and ZEN might reach the human food chain via them.

Although the ZEN+ contaminated organs were positively associated with damages and oxidative imbalance, this condition is not necessarily related to the biological effects of the ZEN alone contamination via poisoned food. In fact, the toxicity of mycotoxins should be addressed in the context of their mixture, and the mild fibrosis detected, as well as inflammation found in very few samples out of the total, could also be due to other environmental factors related to the wildlife [50,51]. Since wild boars inhabit wide open areas, it is impossible to estimate how much ZEN and/or other environmental pollutants they consume daily. Consequently, the human intake of contaminated meat cannot be precisely assessed. In addition, the tested samples belonging to the ZEN− and ZEN+ groups are limited to a small geographical area, which is the province of Avellino. Here, and in other areas of Italy, some traditional meat preparations are based on the liver and kidneys. In this way, ZEN can enter the human food chain, posing a health risk. In fact, the ZEN concentrations in the analyzed organs were higher than those observed by Pałubicki et al. [21], and this condition may suggest a wider ZEN contamination in the sampling area. Not surprisingly, the amounts of ZEN in the livers of farm pigs fed a regulated diet are much lower [52]. 

In the light of these data, and despite the limitations of the study, it is clear that preventive measures against this toxin need to be taken. Due to the detrimental effects of ZEN, the European Union has set certain limits via regulations on their content in food and feed to preserve the health of both citizens and animals (EU No. 2023/915). The International Committee of Risk Evaluation to Mycotoxin Exposure was also concerned about ZEN contamination [53]. However, there are no limits set for game meat consumption and, due to the climatic changes we are currently experiencing with warm temperatures and high humidity, are favoring the development of mycotoxins [54]. Hence, it is evident from the present study that risk assessments concerning the effects of ZEN in livers, kidneys, and muscles may not be restricted to livestock’s direct consumption of grain-based food and feeds but should include the analysis of mycotoxins, including ZEN, also regarding game meat consumption. 

## 5. Conclusions

The results of this study showed the involvement (in terms of OS) of the livers and, although to a lesser extent, kidneys in ZEN-contaminated wild boars. The increased lipid peroxidation and the decreased activity of endogenous antioxidants, as well as morphological alterations, indicated a consistent pattern of potential modifications that affect ZEN-contaminated wild boars. Although further evidence (based on experimental studies) is needed, strengthening mycotoxicosis surveillance in wildlife products for human consumption may be beneficial for human health.

## Data Availability

The data presented in this study are available in the article.
